# Slit2 signaling stimulates Ewing sarcoma growth

**DOI:** 10.18632/genesandcancer.227

**Published:** 2022-12-14

**Authors:** Kruthi Suvarna, Panneerselvam Jayabal, Xiuye Ma, Yuzuru Shiio

**Affiliations:** ^1^Greehey Children’s Cancer Research Institute, The University of Texas Health Science Center, San Antonio, TX 78229-3900, USA; ^2^Cancer Therapy and Research Center, The University of Texas Health Science Center, San Antonio, TX 78229-3900, USA; ^3^Department of Biochemistry and Structural Biology, The University of Texas Health Science Center, San Antonio, TX 78229-3900, USA

**Keywords:** cdc42, Ewing sarcoma, EWS::FLI1, Slit2, Robo

## Abstract

Ewing sarcoma is a cancer of bone and soft tissue in children driven by EWS::ETS fusion, most commonly EWS::FLI1. Because current cytotoxic chemotherapies are not improving the survival of those with metastatic or recurrent Ewing sarcoma cases, there is a need for novel and more effective targeted therapies. While EWS::FLI1 is the major driver of Ewing sarcoma, EWS::FLI1 has been difficult to target. A promising alternative approach is to identify and target the molecular vulnerabilities created by EWS::FLI1.

Here we report that EWS::FLI1 induces the expression of Slit2, the ligand of Roundabout (Robo) receptors implicated in axon guidance and multiple other developmental processes. EWS::FLI1 binds to the Slit2 gene promoter and stimulates the expression of Slit2. Slit2 inactivates cdc42 and stabilizes the BAF chromatin remodeling complexes, enhancing EWS::FLI1 transcriptional output. Silencing of Slit2 strongly inhibited anchorage-dependent and anchorage-independent growth of Ewing sarcoma cells. Silencing of Slit2 receptors, Robo1 and Robo2, inhibited Ewing sarcoma growth as well. These results uncover a new role for Slit2 signaling in stimulating Ewing sarcoma growth and suggest that this pathway can be targeted therapeutically.

## INTRODUCTION

Ewing sarcoma is an aggressive cancer of bone and soft tissue in children. The prognosis of those with metastatic or recurrent Ewing sarcoma cases continue to be poor. Ewing sarcoma is characterized by the reciprocal chromosomal translocation generating a fusion oncogene between EWS and an Ets family transcription factor, most commonly FLI1 [1–3]. EWS::FLI1 translocation accounts for 85% of Ewing sarcoma cases. The EWS::FLI1 gene product regulates the expression of a number of genes important for cancer progression [4], can transform mouse cells [5, 6], and is necessary for proliferation and tumorigenicity of Ewing sarcoma cells [1–3]. Therefore, EWS::FLI1 is considered a causative oncoprotein for Ewing sarcoma. Concerning the mechanism of gene activation by EWS::FLI1, EWS::FLI1 recruits the BAF chromatin remodeling complexes to the target genes to activate their expression [7].

Slits are the ligands of Roundabout (Robo) receptors. Slit was originally identified through a genetic screen for mutations affecting the larval cuticle patterns in Drosophila [8]. Most vertebrates harbor three Slit genes, encoding secreted proteins of approximately 200 kDa. Slit - Robo signaling plays a pivotal role in axon guidance [9, 10]. Binding of Slit ligand to Robo1/2 receptor on the surface of axons results in repulsion of the axons through inactivation of a Rho family G protein, cdc42 [11–14]. Slit – Robo signaling has also been implicated in angiogenesis and organogenesis of kidney, heart, mammary gland, and diaphragm [9, 10].

Using secretome proteomics, we found that EWS::FLI1 induces the expression of Slit2. We demonstrate that EWS::FLI1 binds to the gene promoter of Slit2 and activates its expression. Silencing of Slit2 resulted in activation of cdc42, reduced protein levels of the subunits of the BAF chromatin remodeling complexes, and suppression of EWS::FLI1 target genes. Slit2 silencing strongly inhibited both anchorage-dependent and anchorage-independent growth as well as sphere formation of Ewing sarcoma cells. These results suggest that Ewing sarcoma depends on Slit2 signaling, which can be targeted therapeutically.

## RESULTS AND DISCUSSION

### EWS::FLI1 activates Slit2 expression in Ewing sarcoma

**Figure 1:**
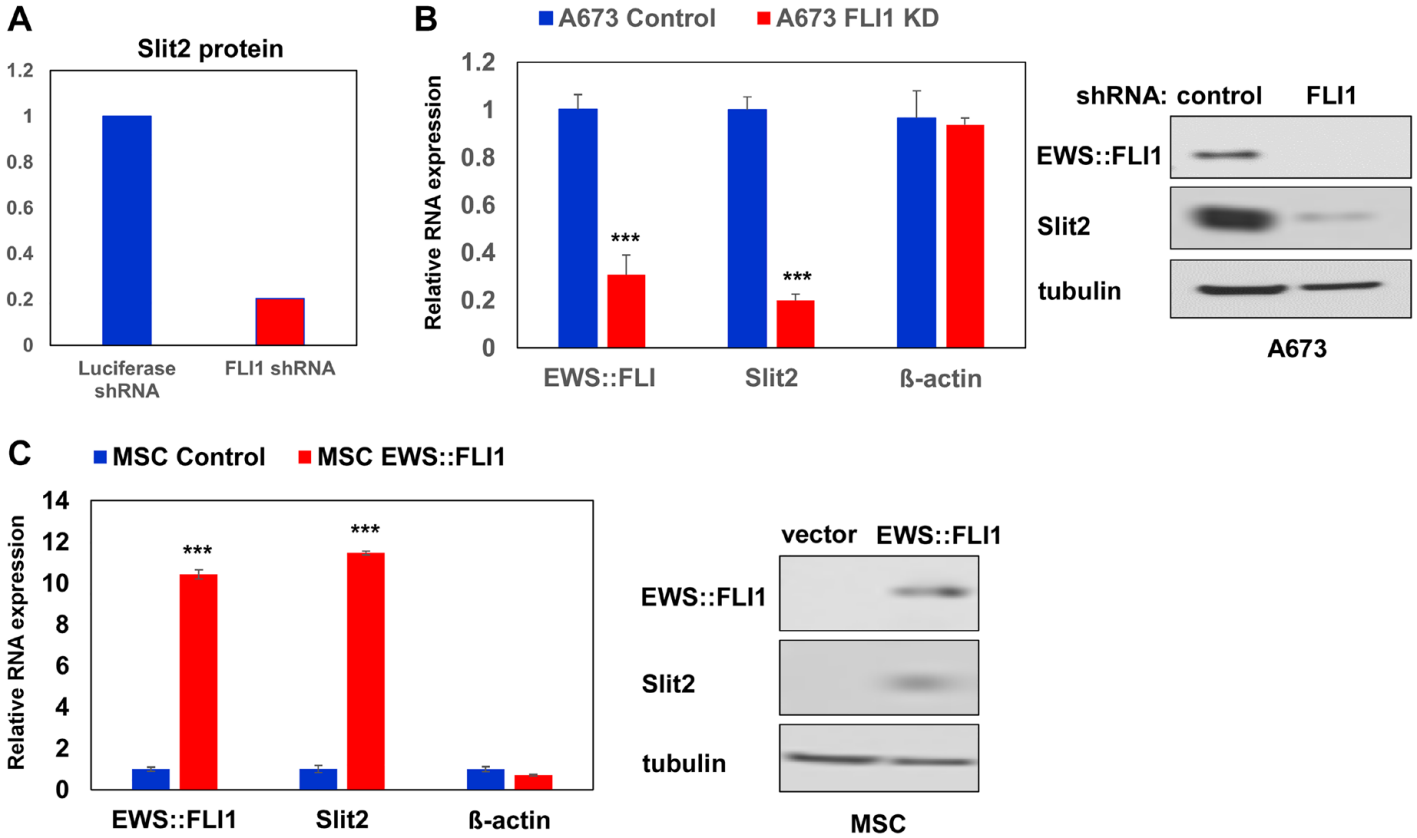
EWS::FLI1 induces Slit2 expression. (**A**) Reduced Slit2 protein levels in A673 cell secretome upon EWS::FLI1 silencing. The quantification is based on spectral counting by mass spectrometry. (**B**) EWS::FLI1 silencing in A673 cells results in reduced Slit2 expression levels. A673 cells were infected with lentiviruses expressing a shRNA against FLI1 C-terminal region or control shRNA and were selected with 2 μg/ml puromycin for 2 days. The EWS::FLI1, Slit2, and β-actin mRNA levels were examined by quantitative real-time RT-PCR (qRT-PCR; left). Whole cell lysates were prepared and immunoblotting was performed using antibodies against FLI1 C-terminus, Slit2, and tubulin (right). ^***^p < 0.001 compared with control shRNA-expressing cells. (**C**) EWS::FLI1 expression in human mesenchymal stem cells results in increased Slit2 expression levels. Human mesenchymal stem cells were infected with lentiviruses expressing EWS::FLI1 or empty vector and were selected with 2 μg/ml puromycin for 2 days. The EWS::FLI1, Slit2, and β-actin mRNA levels were examined by qRT-PCR (left). Whole cell lysates were prepared and immunoblotting was performed using antibodies against FLI1 C-terminus, Slit2, and tubulin (right). ^***^p < 0.001 compared with control shRNA-expressing cells.

**Figure 2:**
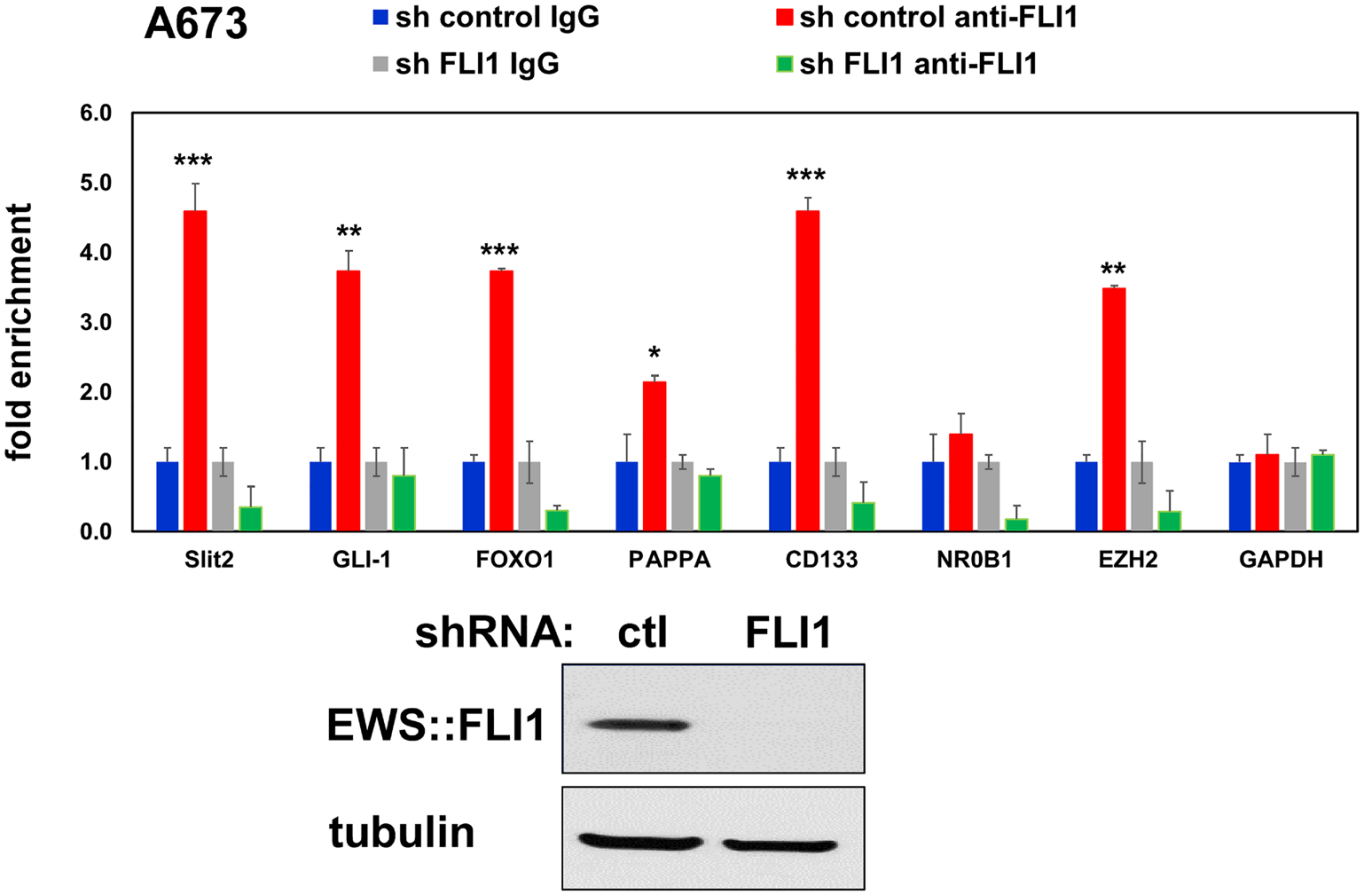
EWS::FLI1 binds to the Slit2 gene promoter in Ewing sarcoma cells. The binding of EWS::FLI1 to the Slit2 promoter was examined by chromatin immunoprecipitation in A673 Ewing sarcoma cells (top). The specificity of the binding was verified by EWS::FLI1 silencing. GLI-1, FOXO1, PAPPA, CD133, NR0B1, and EZH2 are known EWS::FLI1 target genes. GAPDH serves as a negative control. ^*^p < 0.05, ^**^p < 0.01, ^***^p < 0.001 The silencing of EWS-FLI was verified by immunoblotting (bottom).

**Figure 3:**
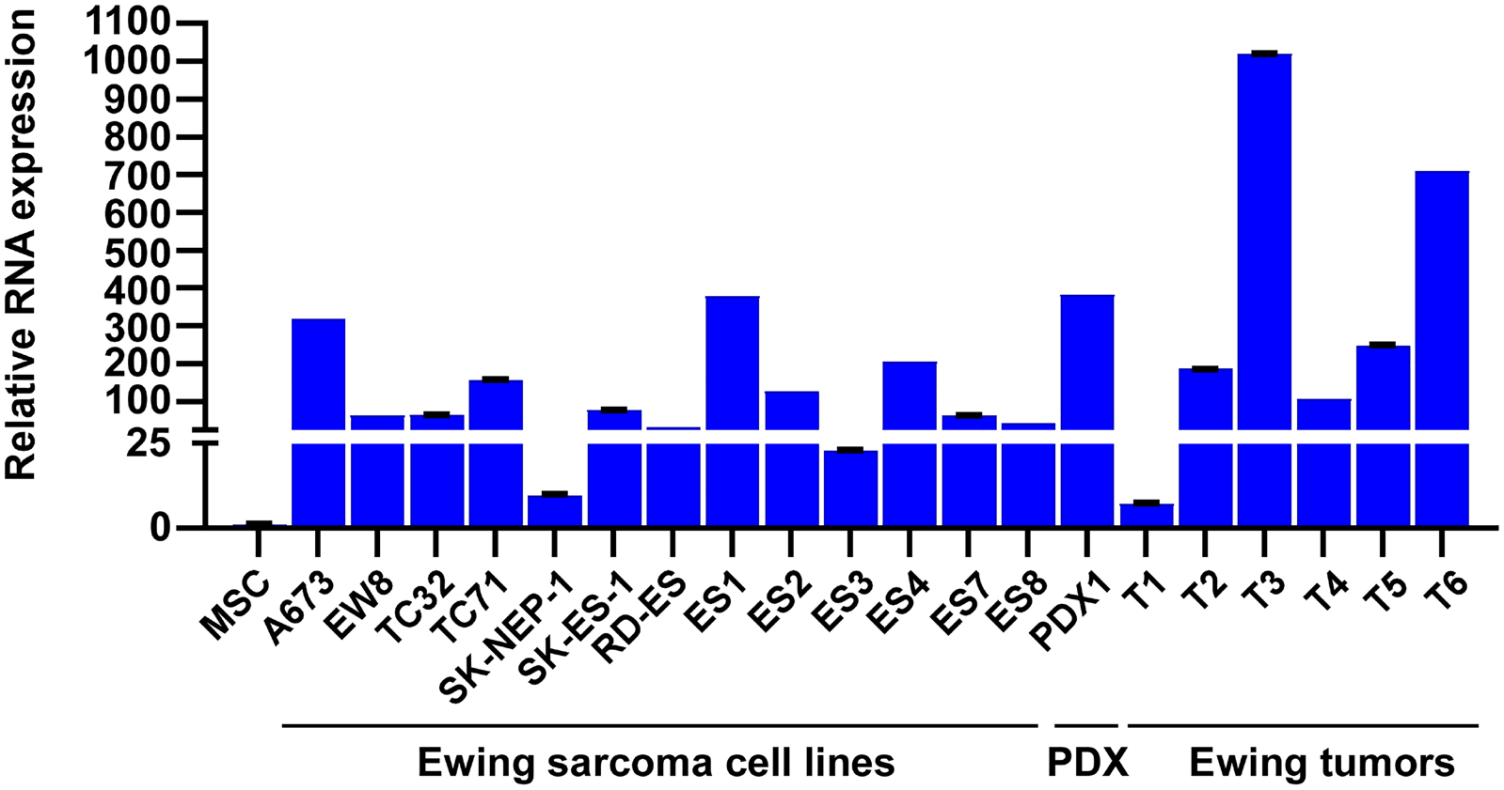
Slit2 RNA expression in Ewing sarcoma cell lines and tumors. The Slit2 mRNA expression in thirteen Ewing sarcoma cell lines, one Ewing sarcoma patient tumor-derived cells, six Ewing sarcoma tumor samples, and human mesenchymal stem cells was examined by qRT-PCR. All RNA levels were normalized to the RNA levels in human mesenchymal stem cells.

We have previously examined the impact of EWS::FLI1 on Ewing sarcoma cell secretome by silencing EWS::FLI1 using a shRNA against FLI1 C-terminal region in A673 Ewing sarcoma cells and analyzing the proteins secreted in the conditioned medium [15]. One of the high-confidence proteins that displayed a significant alteration in abundance upon EWS::FLI1 silencing was Slit2, which exhibited a nearly 5-fold decrease after silencing EWS::FLI1 (Figure 1A). EWS::FLI1 silencing also reduced Slit2 RNA and protein levels in A673 cells (Figure 1B). Conversely, exogenous expression of EWS::FLI1 in human mesenchymal stem cells, the putative cells of origin of Ewing sarcoma [1–3], induced Slit2 expression (Figure 1C). Using chromatin immunoprecipitation, we detected the binding of endogenous EWS::FLI1 to the Slit2 gene promoter in A673 Ewing sarcoma cells, and this binding was abolished by shRNA-mediated silencing of EWS::FLI1 (Figure 2). These results indicate that Slit2 is a novel direct transcriptional target of EWS::FLI1. Consistent with the activation of Slit2 gene transcription by EWS::FLI1, Ewing sarcoma tumors and cell lines expressed much higher levels of Slit2 mRNA than human mesenchymal stem cells (Figure 3).

### Ewing sarcoma depends on Slit2

**Figure 4:**
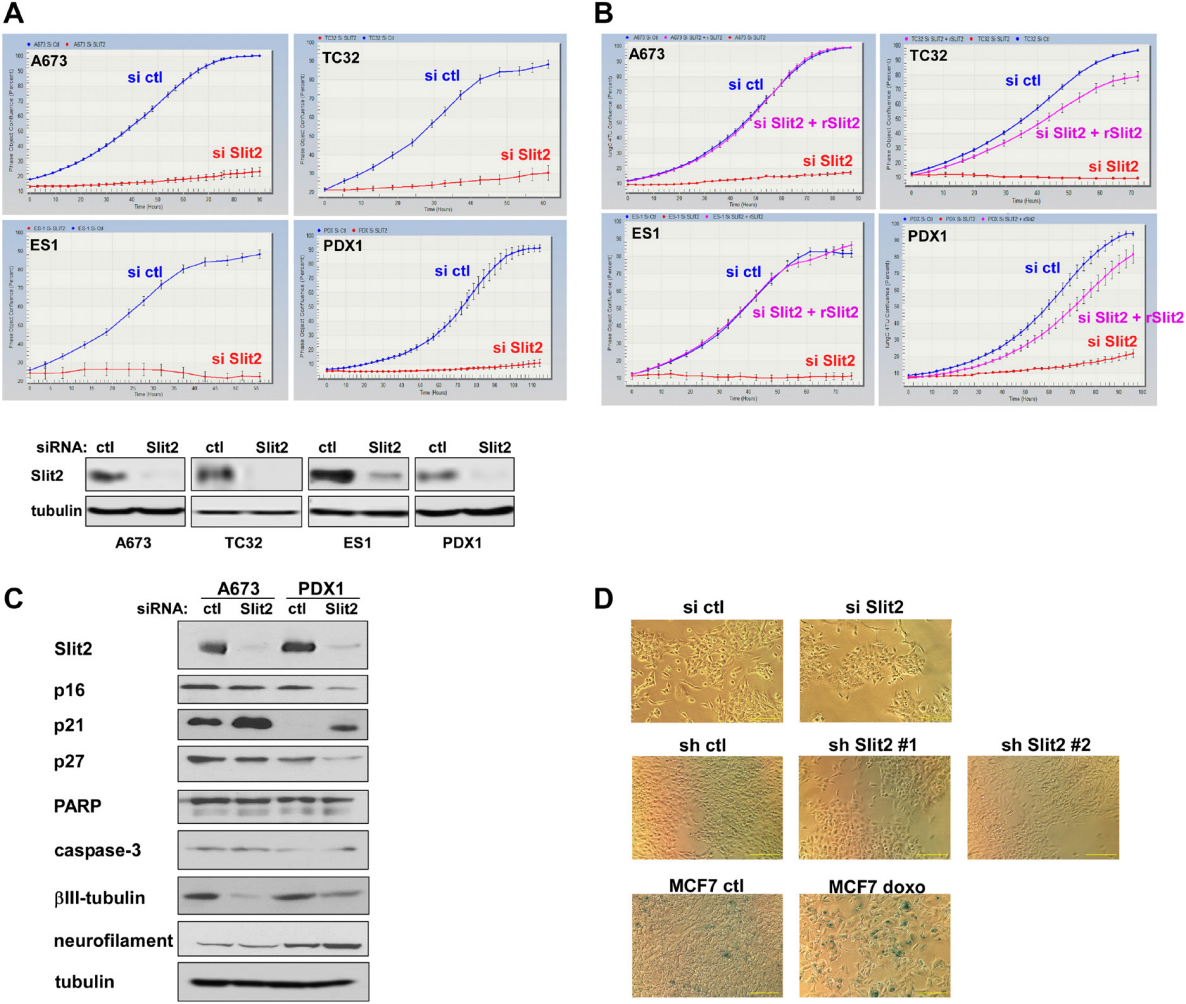
Ewing sarcoma depends on Slit2. (**A**) Slit2 silencing inhibits Ewing sarcoma proliferation. Slit2 was silenced by siRNAs in four different Ewing sarcoma cells and cell proliferation was assessed by the IncuCyte live-cell imaging system. Slit2 silencing was verified by immunoblotting (bottom). (**B**) Recombinant Slit2 rescues proliferation arrest induced by Slit2 silencing. Ewing sarcoma cells were transfected with Slit2 or control siRNAs and were treated with or without recombinant Slit2 (250 ng/ml). Cell proliferation was assessed by IncuCyte. (**C**) Slit2 silencing does not induce apoptosis or neuronal differentiation. Slit2 was silenced in A673 and PDX1 cells and the expression of the indicated protein was examined by immunoblotting. (**D**) Slit2 silencing does not induce senescence. Slit2 was silenced by siRNA (top) or by two different shRNAs (middle) in A673 cells and cells were stained for senescence-associated beta-galactosidase. MCF7 breast cancer cells were treated with 1 μM doxorubicin for 2 hours and 4 days later, stained for senescence-associated beta-galactosidase (bottom). Doxorubicin-treated MCF7 cells displayed roust senescence-associated beta-galactosidase activity as we reported previously [30] whereas Slit2-silenced A673 cells did not detectably display senescence-associated beta-galactosidase activity. Scale bars: 100 μm.

To dissect the role of Slit2 in Ewing sarcoma, we tested the effects of manipulation of Slit2 expression. Silencing of Slit2 by a pool of siRNAs (Dharmacon siGENOME Human SLIT2 siRNA SMARTPool, #M-019853-01-0005) strongly inhibited the proliferation of all three Ewing sarcoma cell lines tested (A673, TC32, and ES1) (Figure 4A). We also dissociated a Ewing sarcoma patient-derived xenograft tumor to cells (PDX1) and found that the proliferation of these patient tumor-derived cells is strongly suppressed by Slit2 silencing (Figure 4A). Furthermore, the proliferation inhibition by Slit2 silencing was completely rescued by the addition of purified recombinant Slit2 protein (R&D Recombinant Human Slit2 (aa 26-1118) Protein, CF, #8616-SL-050) to the culture medium (Figure 4B). These results indicate that Ewing sarcoma cell lines and Ewing sarcoma patient tumor-derived cells both depend on extracellular Slit2.

Slit2 silencing induced the CDK inhibitor, p21, in A673 and PDX1 cells (Figure 4C). Slit2 silencing did not induce apoptosis (cleavage of PARP or caspase-3; Figure 4C), neuronal differentiation (βIII-tubulin and neurofilament L; Figure 4C), or senescence (senescence-associated beta-galactosidase; Figure 4D).

### Slit2 signaling inactivates cdc42 in Ewing sarcoma

**Figure 5:**
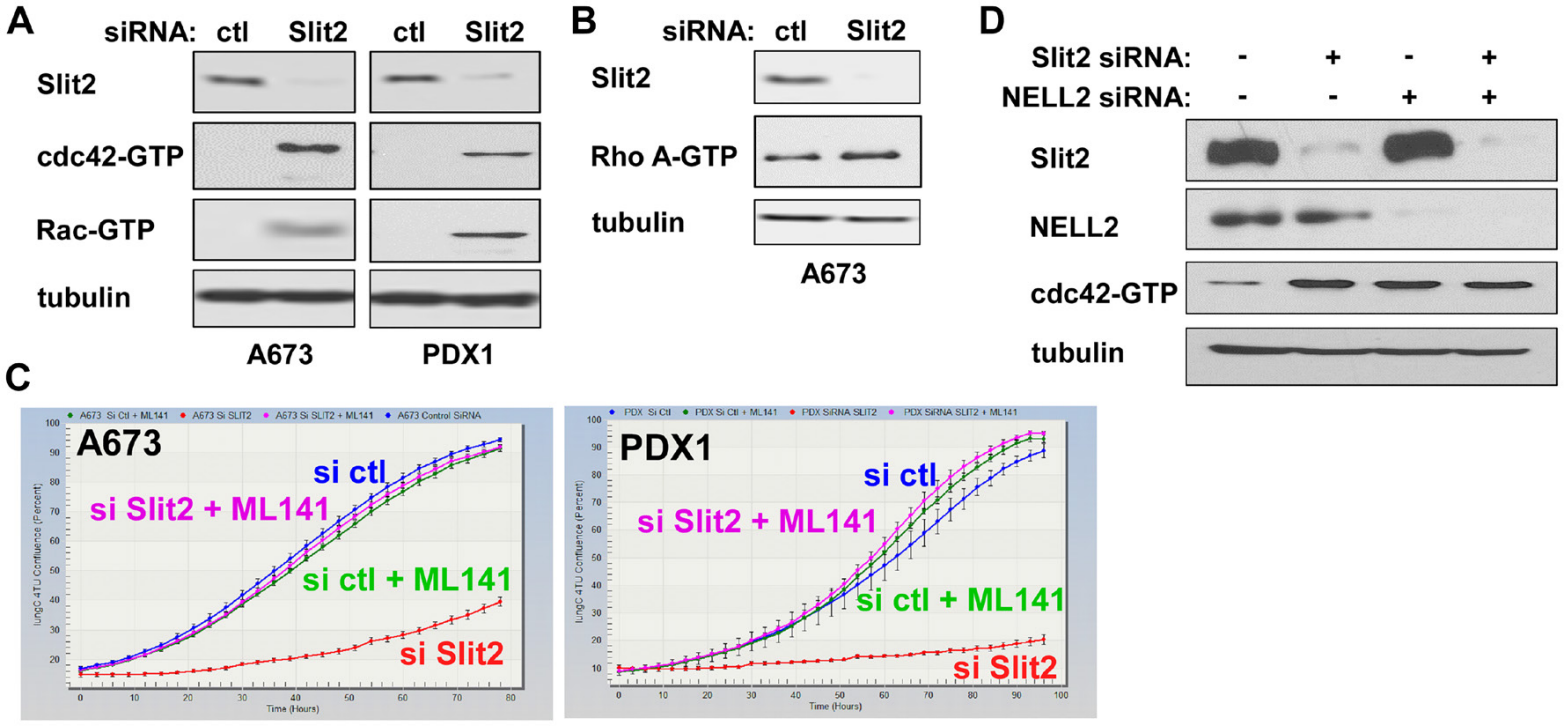
Slit2 signaling inhibits cdc42 in Ewing sarcoma. (**A**) Slit2 silencing activates cdc42 and Rac in Ewing sarcoma. A673 and PDX1 cells were transfected with Slit2 siRNAs or control siRNAs. Two days after transfection, the levels of GTP-bound, active cdc42 and Rac were examined by GST-PAK1 pull-down of whole cell lysate followed by anti-cdc42 and Rac immunoblotting. (**B**) Slit2 silencing barely affects active Rho A levels in Ewing sarcoma. A673 cells were transfected with Slit2 siRNAs or control siRNAs. Two days after transfection, the levels of GTP-bound, active Rho A were examined GST-Rhotekin-RBD pull-down of whole cell lysate followed by anti-Rho A immunoblotting. (**C**) Growth arrest induced by Slit2 silencing can be rescued by a cdc42 inhibitor, ML141. A673 and PDX1 cells were transfected with Slit2 siRNAs or control siRNAs and were treated with or without 5 μM ML141. Cell proliferation was assessed by IncuCyte. (**D**) Both Slit2 and NELL2 are necessary to suppress cdc42 in Ewing sarcoma. A673 cells were transfected with Slit2 siRNAs and/or NELL2 siRNAs as indicated. Two days after transfection, the levels of active cdc42 were examined.

Slit2 belongs to the Slit family of secreted proteins that are implicated in axon guidance during development [9, 10]. Slit signaling inactivates Rho family G proteins, primarily cdc42, resulting in axon repulsion [11–14]. We therefore tested how Slit2 affects the levels of active Rho family G proteins in Ewing sarcoma. Using the pull-down of cell lysate with GST-PAK1, which selectively interacts with GTP-bound active cdc42 and active Rac, we showed that Slit2 silencing strongly increases the levels of active cdc42 and active Rac in A673 Ewing sarcoma cells and patient tumor-derived cells (Figure 5A). We also used the cell lysate pull-down with GST-Rhotekin Rho-binding domain (RBD), which selectively interacts with GTP-bound active Rho A, and found that Slit2 silencing barely affects the levels of active Rho A in Ewing sarcoma (Figure 5B). Importantly, the proliferation inhibition of Ewing sarcoma cells by Slit2 silencing was completely rescued by a selective inhibitor of cdc42, ML141 (Figure 5C), suggesting Slit2 signaling normally maintains the proliferation of Ewing sarcoma cells by inactivation of cdc42.

We recently reported that Ewing sarcoma depends on the autocrine signaling mediated by NELL2, which maintains Ewing sarcoma growth through inactivation of cdc42 [15]. We found that silencing of Slit2 and NELL2, singly or in combination, induced similar levels of active cdc42 (Figure 5D), suggesting that both Slit2 signaling and NELL2 signaling are necessary to inactivate cdc42 in Ewing sarcoma.

### Slit2 – cdc42 signaling regulates the BAF chromatin remodeling complexes and the transcriptional output of EWS::FLI1

**Figure 6:**
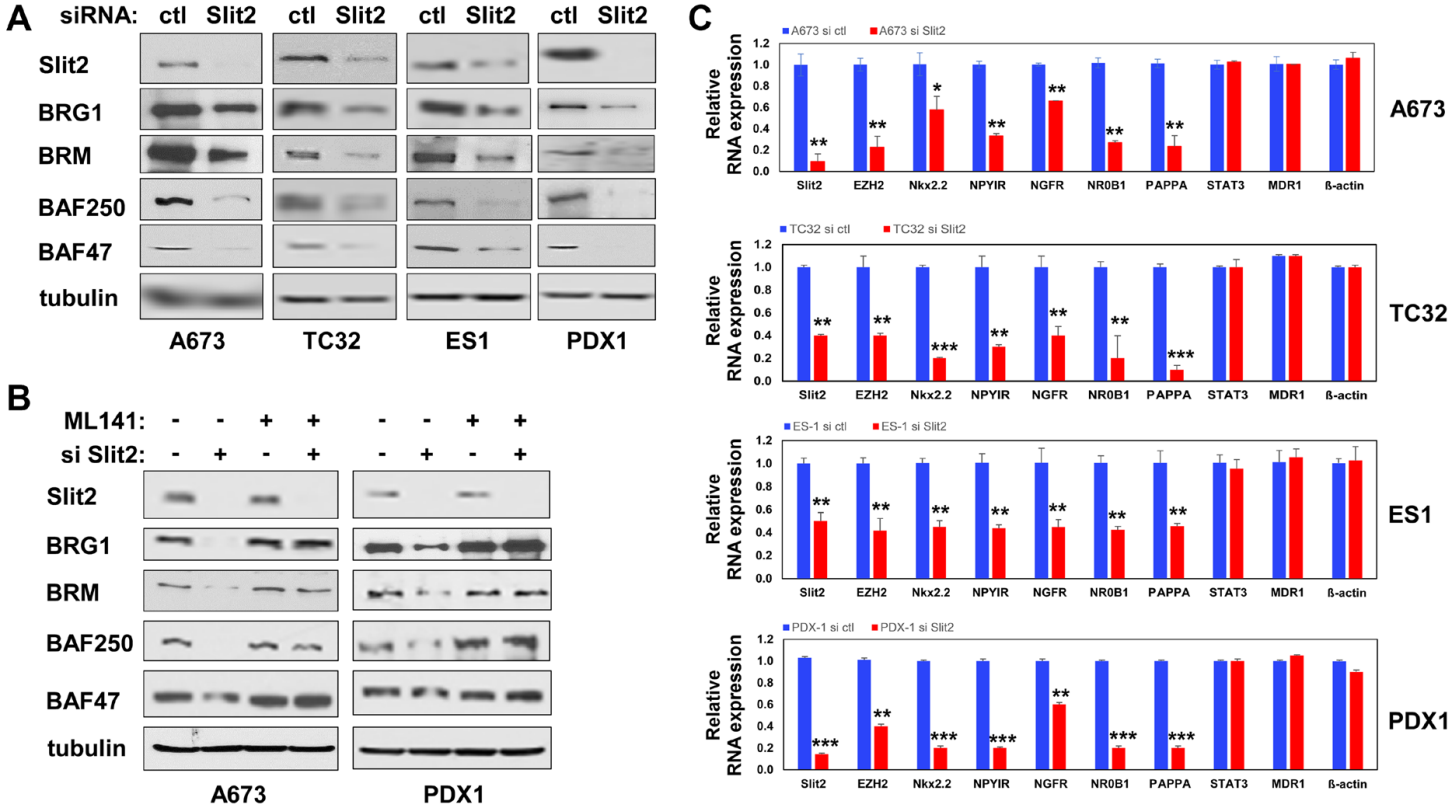
Slit2 signaling stabilizes BAF complex subunits and stimulates EWS::FLI1 transcriptional output. (**A**) Slit2 silencing reduces the protein levels of BAF complex subunits. A673, TC32, ES1, and PDX1 Ewing sarcoma cells were transfected with Slit2 siRNAs or control siRNAs and the levels of indicated BAF subunits were examined by immunoblotting. (**B**) The cdc42 inhibitor ML141 restores the protein levels of BAF subunits in Slit2-silenced cells. Ewing sarcoma cells were transfected with Slit2 siRNAs or control siRNAs and were treated with or without 5 μM ML141. The levels of BAF subunits were assessed by immunoblotting. (**C**) Slit2 silencing reduces EWS::FLI1 target gene expression in Ewing sarcoma. A673, TC32, ES1, and PDX1 Ewing sarcoma cells were transfected with Slit2 siRNAs or control siRNAs and the RNA expression of indicated genes was examined by qRT-PCR and is presented after normalization to the levels in control siRNAs transfected cells (blue). The expression of EWS::FLI1 target genes is reduced in Slit2-silenced cells (red). ^*^p < 0.05, ^**^p < 0.01, ^***^p < 0.001.

We have previously demonstrated that cdc42 plays a critical inhibitory role in Ewing sarcoma by destabilizing the BAF chromatin remodeling complexes and suppressing EWS::FLI1 target gene expression [15]. We therefore assessed how Slit2 signaling affects the levels of BAF complex subunits in Ewing sarcoma. We found that Slit2 silencing reduces the protein levels of key BAF subunits, BRG1, BRM, BAF250, and BAF47 (Figure 6A), which we demonstrated to be destabilized by cdc42 [15]. The reduced protein levels of these BAF subunits upon Slit2 silencing were rescued by the cdc42 inhibitor, ML141, (Figure 6B). These results suggest that Slit2 – cdc42 signaling regulates the protein levels of these key BAF subunits in Ewing sarcoma.

The Ewing sarcoma fusion oncoprotein, EWS::FLI1, recruits the BAF chromatin remodeling complexes to activate its target genes [7]. Consistent with this, Slit2 silencing suppressed the expression of EWS::FLI1 target genes in Ewing sarcoma, including EZH2 [16, 17], NKX2.2 [18], NPY1R [19], NGFR [20], NR0B1 [21], and PAPPA [22] (Figure 6C).

### Slit2 receptors, Robo1 and Robo2, regulate cdc42 and proliferation in Ewing sarcoma

**Figure 7:**
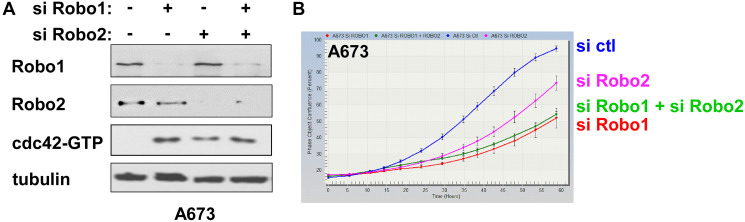
The silencing of Robo1/2 receptors activates cdc42 and inhibits Ewing sarcoma cell proliferation. (**A**) The silencing of Robo1/2 activates cdc42 and Rac in Ewing sarcoma. A673 cells were transfected with Robo1 siRNAs, Robo2 siRNAs and/or control siRNAs as indicated. Two days after transfection, the levels of GTP-bound, active cdc42 were examined by GST-PAK1 pull-down of whole cell lysate followed by anti-cdc42 immunoblotting. The silencing of Robo1 and Robo2 was verified by immunoblotting. (**B**) The silencing of Robo1/2 inhibits Ewing sarcoma cell proliferation. A673 cells were transfected with Robo1 siRNAs, Robo2 siRNAs and/or control siRNAs as indicated and cell proliferation was assessed by IncuCyte.

Slit2 is a ligand of transmembrane receptors, Robo1 and Robo2, and Slit – Robo signaling inactivates cdc42, leading to axon repulsion [14]. The silencing of Robo1 and/or Robo2 resulted in activation of cdc42 in Ewing sarcoma cells (Figure 7A). Furthermore, Robo1/Robo2 silencing inhibited Ewing sarcoma cell proliferation (Figure 7B). These results suggest that Slit2 – Robo1/2 signaling inhibits cdc42 and stimulates proliferation in Ewing sarcoma.

### Slit2 signaling stimulates the transformed phenotype of Ewing sarcoma cells

**Figure 8:**
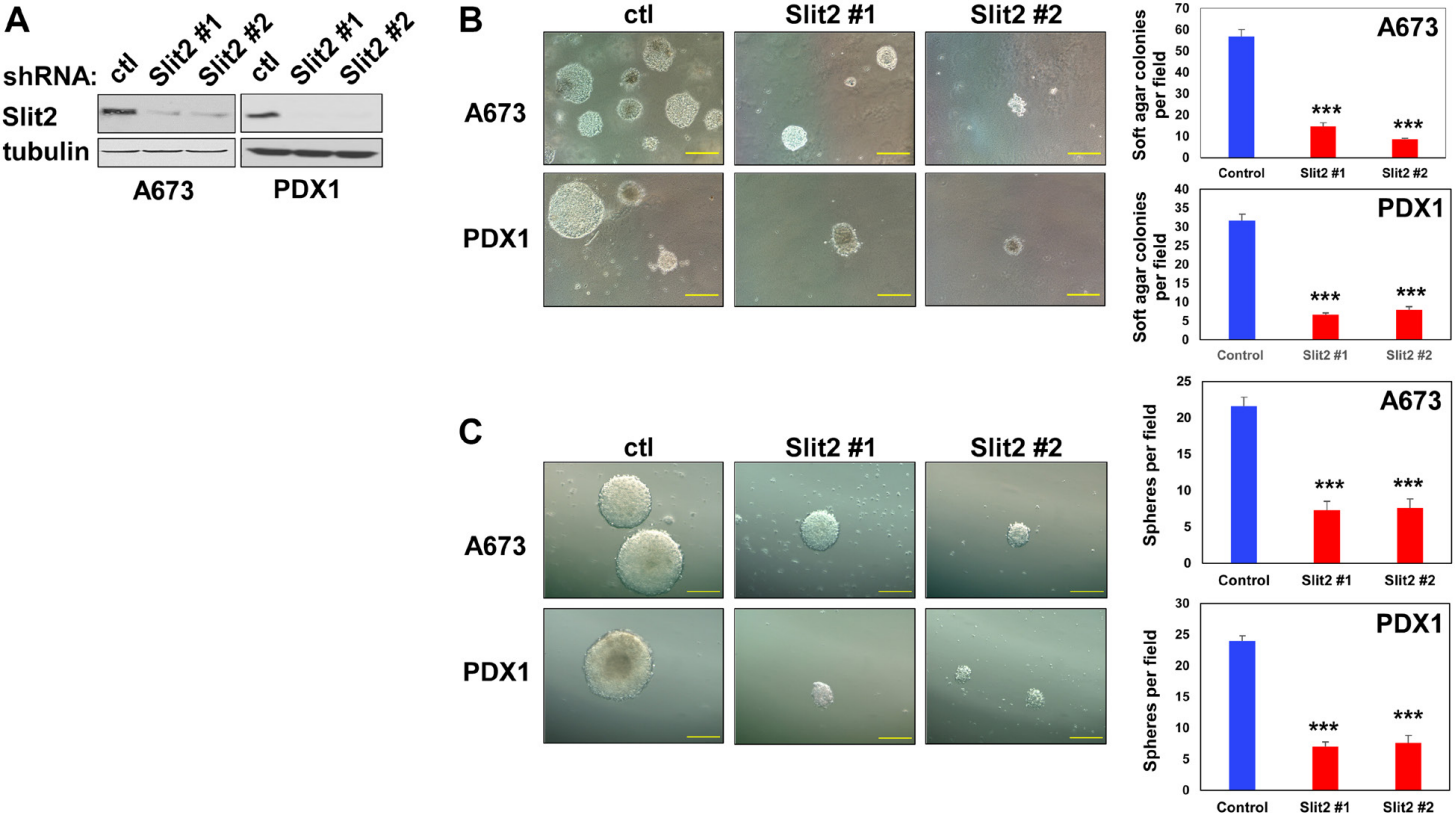
Slit2 signaling stimulates the transformed phenotype of Ewing sarcoma cells. (**A**) shRNA-mediated silencing of Slit2. A673 and PDX1 cells were infected with lentiviruses expressing two different shRNAs against Slit2 or control shRNA and were selected with 2 μg/ml puromycin. Four days after infection, the silencing of Slit2 was verified by immunoblotting. (**B**) The silencing of Slit2 impairs anchorage-independent growth. Cells in (A) were plated in semi-solid medium. Two weeks after culture, colonies were counted and photographed. ^***^p < 0.001 Scale bars: 100 mm. (**C**) The silencing of Slit2 impairs sphere formation. Cells in (A) were plated in ultra-low attachment 6-well plates. Two weeks after culture, spheres were counted and photographed. ^***^p < 0.001 Scale bars: 100 mm.

Cancer cells are characterized by deregulation of normal growth control, transformation. To assess the role of Slit2 signaling in the transformed phenotype of Ewing sarcoma cells, we first assessed the effect of Slit2 silencing on anchorage-independent growth. A673 and PDX1 Ewing sarcoma cells were infected with lentiviruses expressing two different shRNAs against Slit2 or control scrambled shRNA and were selected by puromycin. Slit2 shRNAs efficiently silenced Slit2 protein expression in both Ewing sarcoma cells (Figure 8A). When the shRNA-expressing Ewing sarcoma cells were grown in soft agar, we found that Slit2 silencing strongly inhibits colony formation in soft agar (anchorage-independent growth) (Figure 8B). Sphere formation assays are commonly used to identify cancer stem-like cells [23]. We found that shRNA-mediated silencing of Slit2 results in significant suppression of sphere formation by A673 and PDX1 Ewing sarcoma cells (Figure 8C). These results extend the strong anti-proliferative effect of Slit2 silencing observed in anchorage-dependent culture (Figure 4A) and suggest that Slit2 signaling normally stimulates the transformed phenotype of Ewing sarcoma cells.

Slit – Robo signaling has been implicated in multiple developmental processes such as axon guidance, angiogenesis, and organogenesis of kidney, heart, mammary gland, and diaphragm [9, 10]. This study extended the role of Slit - Robo signaling to growth control of Ewing sarcoma cells. We found that Slit2 is a direct transcriptional activation target of EWS::FLI1 and that Ewing sarcoma depends on the autocrine signaling mediated by Slit2. Slit2 signaling inactivates cdc42, stabilizes the BAF chromatin remodeling complex subunits, and maintains the expression of EWS::FLI1 target genes. The silencing of Slit2 severely inhibited anchorage-dependent and anchorage-independent growth as well as sphere formation of Ewing sarcoma cells.

Previous studies implicated Slit – Robo signaling in a variety of tumors. There are a number of reports on reduced expression of Slit ligands and Robo receptors due to promoter hypermethylation in tumors (such as lung cancer, breast cancer, cervical cancer, and glioblastoma) compared with normal tissues, suggesting a tumor suppressor role for this pathway [10, 24]. By contrast, overexpression of Slit or Robo was observed in prostate cancer [25] and Slit - Robo signaling was shown to promote melanoma tumor angiogenesis [26]. The latter findings suggest a tumor-promoting role for Slit - Robo signaling. These studies suggest that Slit-Robo signaling plays both tumor-suppressing and tumor-promoting roles depending on tumor types. The present study demonstrates that Slit-Robo signaling plays a tumor-promoting role in Ewing sarcoma.

We recently reported that Ewing sarcoma depends on the autocrine signaling mediated by a secreted protein NELL2 and its receptor Robo3 [15]. Like Slit2, NELL2 is a direct transcriptional activation target of EWS::FLI1 [15]. The binding of NELL2 to Robo3 results in inactivation of cdc42, stabilization of the BAF chromatin remodeling complexes, and stimulation of EWS::FLI1 transcriptional output [15]. The silencing of NELL2 or Robo3 severely impairs Ewing sarcoma cell growth [15]. The biological roles and the molecular mechanisms of Slit2 - Robo1/2 signaling in Ewing sarcoma uncovered by the present study parallel those of NELL2 - Robo3 signaling. The identification of two parallel signaling pathways that inactivate cdc42 in Ewing sarcoma underscores the critical inhibitory role for cdc42 in this cancer.

The EWS::FLI1 fusion oncoprotein is the primary driver of Ewing sarcoma. Thus, targeting EWS::FLI1 is a Rational approach to treat Ewing sarcoma. Despite many attempts, however, EWS::FLI1-targeted therapy has not been developed to date and EWS::FLI1 continues to be “the perfect target without a therapeutic agent [27].” One way to resolve this conundrum is to identify and target the vulnerabilities created by EWS::FLI1. The Slit2 and NELL2 autocrine loops represent such vulnerabilities created by EWS::FLI1.

Ewing sarcoma’s dependence on Slit2 signaling provides an excellent opportunity for therapeutic targeting. While a pharmacological inhibitor for Slit – Robo signaling is not yet available, a radioactively labeled anti-Robo1 monoclonal antibody was shown to suppress xenograft tumor growth of hepatocellular carcinoma cells [28] and small cell lung cancer cells [29], suggesting that it is feasible to target the Slit – Robo pathway. Therapeutic targeting of Slit2 - Robo1/2 signaling singly or in combination with that of NELL2 - Robo3 signaling in Ewing sarcoma warrants further investigations.

## MATERIALS AND METHODS

### Cell culture

A673 and 293T cells were cultured in Dulbecco’s modified Eagle’s medium (DMEM) supplemented with 10% fetal bovine serum. EW8, TC32, TC71, ES1, ES2, ES3, ES4, ES7, ES8, and RD-ES cells were cultured in RPMI-1640 medium supplemented with 10% fetal bovine serum. SK-NEP-1 and SK-ES-1 cells were cultured in McCoy’s 5a medium supplemented with 15% fetal bovine serum. A673, SK-NEP-1, SK-ES-1, RD-ES, and 293T cells were from ATCC. TC71 cells were from the Coriell Institute for Medical Research. EW8 and TC32cells were from Dr. Patrick Grohar. ES1, ES2, ES3, ES4, ES7, and ES8 cells were from Dr. Peter Houghton. The cell lines were STR-authenticated and were routinely tested for the absence of mycoplasma. Cord blood-derived human mesenchymal stem cells were purchased from Vitro Biopharma (Golden, CO, USA) and were cultured in low-serum MSC-GRO following the manufacturer’s procedure. PDX1 cells dissociated from a Ewing sarcoma patient-derived xenograft tumor (NCH-EWS-1) were cultured in DMEM/F-12 medium supplemented with 10% FBS [15]. Calcium phosphate co-precipitation was used for transfection of 293T cells. Lentiviruses were prepared by transfection in 293T cells following System Biosciences’ protocol and the cells infected with lentiviruses were selected with 2 μg/ml puromycin for 48 hours as described [30, 31]. The target sequences for shRNAs are as follows: FLI1 C-terminus shRNA, AACGATCAGTAAGAATACAGAGC; luciferase shRNA, GCACTCTGATTGACAAATACGATTT; Slit2 shRNA-1, CCTGGAGCTTTCTCACCATAT; Slit2 shRNA-2, ACTAGAGAGACTGCGTTTAAA; and scrambled shRNA, CCTAAGGTTAAGTCGCCCTCG. The following siRNAs were used: human Slit2 siRNA SMARTPool (M-019853-01-0005, Dharmacon), human Robo1 siRNA SMARTPool (M-011381-00-0005, Dharmacon), human Robo2 siRNA SMARTPool (M-023273-01-0005, Dharmacon), human NELL2 siRNA SMARTPool (M-012185-00-0010, Dharmacon), and Non-Targeting siRNA Pool #2 (D-001206-14-05, Dharmacon). siRNA transfection was performed using Lipofectamine™ RNAiMAX Transfection Reagent (Thermo Fisher). Recombinant Slit2 protein (8616-SL-050; aa 26-1118) was purchased from R&D Systems. ML141 (S7686) was purchased from Selleck Chemicals. Senescence-associated beta-galactosidase staining was performed using Senescence β-Galactosidase Staining Kit (9860, Cell Signaling Technologies).

### Protein sample preparation and proteomic analysis

A673 cells were infected with lentiviruses expressing a shRNA against FLI1 C-terminal region or luciferase (control) and were selected with 2 μg/ml puromycin for 2 days. Cells were washed six times with DMEM without serum. Subsequently, cells were cultured in DMEM without serum for 24 hours and the culture supernatant was harvested. The supernatant was centrifuged, filtered through a 0.45 μm filter (Millipore), and concentrated using a 3,000 Dalton cut-off Amicon Ultra Centrifugal Filter Units (Millipore). The proteins in each sample were fractionated by SDS-PAGE and visualized by Coomassie blue. Each gel lane was divided into six slices, and the proteins in each slice were digested *in situ* with trypsin (Promega modified) in 40 mM NH4HCO3 overnight at 37°C. The resulting tryptic peptides were analyzed by HPLC-ESI-tandem mass spectrometry (HPLC-ESI-MS/MS) on a Thermo Fisher LTQ Orbitrap Velos mass spectrometer. The Xcalibur raw files were converted to mzXML format using ReAdW and were searched against the UniProtKB/Swiss-Prot human protein database using X! Tandem. The X! Tandem search results were analyzed by the Trans-Proteomic Pipeline [32] version 4.3. Peptide/protein identifications were validated by Peptide/ProteinProphet [33, 34].

### RNA samples and quantitative Real-Time PCR

De-identified Ewing sarcoma tumor RNA samples were obtained from the Cooperative Human Tissue Network. Total cellular RNA was isolated using TRIzol reagent (Invitrogen). Reverse transcription was performed using High Capacity cDNA Reverse Transcription Kit (Thermo Fisher) as per manufacturer’s instructions. Quantitative PCR was performed using PowerUp SYBR Green Master Mix (Thermo Fisher) on Applied Biosystems ViiA 7 Real-Time PCR System. Each sample was analyzed in triplicate. The following primers were used: Slit2 forward, 5′-TCCTAACTCCAAAGGGATTCAAATGT-3′, Slit2 Reverse, 5′-GGCTCCGTTTTTACACTTGTTGTCT-3′ NR0B1 forward, 5′-AGGGGACCGTGCTCTTTAAC-3′, NR0B1 reverse, 5′-CTGAGTTCCCCACTGGAGTC-3′; NKX2.2 forward, 5′-CAGCGACAACCCGTACAC-3′, NKX2.2 reverse, 5′-GACTTGGAGCTTGAGTCCTGA-3′; EZH2 forward, 5′-TGGGAAAGTACACGGGGATA-3′, EZH2 reverse, 5′-TATTGACCAAGGGCATTCAC-3′; NPY1R forward, 5′-CCATCGGACTCTCATAGGTTGTC-3′; NPY1R reverse, 5′-GACCTGTACTTATTGTCTCTCATC-3′; NGFR forward, 5′-CCTCATCCCTGTCTATTGCTCC-3′, NGFR reverse, 5′-GTTGGCTCCTTGCTTGTTCTGC-3′; PAPPA forward, 5′-CAGAATGCACTGTTACCTGGA-3′; PAPPA reverse, 5′-GCTGATCCCAATTCTCTTTCA-3′; β-actin forward, 5′-AGAGCTACGAGCTGCCTGAC-3′; β-actin reverse, 5′-AGCACTGTGTTGGCGTACAG-3′; STAT3 forward, 5′-GGCATTCGGGAAGTATTGTCG-3′; STAT3 reverse, 5′-GGTAGGCGCCTCAGTCGTATC-3′; MDR1 forward, 5′-CACGTGGTTGGAAGCTAACC-3′; MDR1 reverse, 5′-GAAGGCCAGAGCATAAGATGC-3′; EWS::FLI1 forward, 5′-GGCAGCAGAACCCTTCTTAT-3′; EWS::FLI1 reverse, 5′-GGCCGTTGCTCTGTATTCTTA-3′; GAPDH forward, 5′-GGTGTGAACCATGAGAAGTATGA-3′; GAPDH reverse, 5′-GAGTCCTTCCACGATACCAAAG-3′.

### Immunoblotting

20 μg of whole-cell lysate was separated by SDS-PAGE and analyzed by immunoblotting as described [15]. The following antibodies were used: rabbit monoclonal anti-Slit2 (47600, Cell Signaling Technologies), rabbit polyclonal anti-FLI1 (ab15289, Abcam); rabbit polyclonal anti-p16 (sc-468, Santa Cruz Biotechnology); mouse monoclonal anti-p21 Waf1/Cip1 (2946, Cell Signaling Technologies); rabbit polyclonal anti-p27 (sc-528, Santa Cruz Biotechnology); rabbit polyclonal anti-PARP (9542, Cell Signaling Technologies); rabbit monoclonal anti-Caspase-3 (9665, Cell Signaling Technologies); rabbit monoclonal anti-Neurofilament-L (2837, Cell Signaling Technologies); rabbit monoclonal anti-β3-Tubulin (5568, Cell Signaling Technologies); mouse monoclonal anti-cdc42 (ACD03, Cytoskeleton); rabbit polyclonal anti-Rac1/2/3 (2465, Cell Signaling Technologies); rabbit polyclonal anti-Rho A (2117, Cell Signaling Technologies); rabbit monoclonal anti-NELL2 (ab181376, Abcam); goat polyclonal anti-BRG1 (A303-877A, Bethyl Laboratories); rabbit polyclonal anti-BRM (A301-014A-T, Bethyl Laboratories); rabbit monoclonal anti-ARID1A/BAF250 (12354, Cell Signaling Technologies); rabbit monoclonal anti-BAF47 (8745, Cell Signaling Technologies); rabbit polyclonal anti-Robo1 (A301-266A, Bethyl Laboratories); mouse monoclonal anti-Robo2 (sc-376177, Santa Cruz Biotechnology); and rabbit monoclonal anti-CD133 (64326, Cell Signaling Technologies). The following HRP-conjugated secondary antibodies were used: goat anti-rabbit (7074) and goat anti-mouse (7076) (Cell Signaling Technologies); donkey anti-goat (A50-201P, Bethyl Laboratories).

### Chromatin immunoprecipitation

Chromatin immunoprecipitation (ChIP) was performed as described [35] using rabbit polyclonal anti-FLI1 antibody (ab15289, Abcam) or control rabbit IgG (ab37415, Abcam). The primer sequences used for ChIP are as follows: Slit2 forward, 5′-GACTAGTGGATATTTCTGCCCG-3′; Slit2 reverse, 5′-CAAACACACATGCACTTCGCTG-3′; FOXO1 forward, 5′-GGAAGAGGTTCCCACGGAGGGCAT-3′; FOXO1 reverse, 5′-CCGGCGACACTTTGTTTACT-3′; GLI-1 forward, 5′-AGAGCCTGGGGGTGAGACAT-3′; GLI-1 reverse, 5′-GCCTCTTCAACTTAACCGCATGA-3′; EZH2 forward, 5′-GACACGTGCTTAGAACTACGAACAG-3′; EZH2 reverse, 5′-TTTGGCTGGCCGAGCTT-3′; NR0B1 forward, 5′-GTTTGTGCCTTCATGGGAAATGGTTATTC-3′; NR0B1 reverse, 5′-CTAGTGTCTTGTGTGTCCCTAGGG-3′; PAPPA forward, 5′-TTAGCTGAAGCCAGCCTTATC-3′; PAPPA reverse, 5′-CCCTTTACCTCTTTCCCTCTTC-3′; GAPDH forward, 5′-TCCTCCTGTTTCATCCAAGC-3′; GAPDH reverse, 5′-TAGTAGCCGGGCCCTACTTT-3′; CD133 P2 forward, 5′-CGACCACAGCGGGAGTAG-3′; CD133 P2 reverse, 5′-GCGAGAGGCTGGGAAGGT-3′.

### Cell proliferation assays

Anchorage-dependent cell proliferation was assessed by IncuCyte live-cell imaging system (Essen BioScience). The IncuCyte system monitors cell proliferation by analyzing the occupied area (% confluence) of cell images over time. At least four fields from four wells were assayed for each experimental condition. The cell seeding density was 2000 cells per well in a 96-well plate. For each assay, biological replicates were performed to confirm the reproducibility of results. Anchorage-independent cell proliferation was evaluated by soft agar colony formation assays. A673 and PDX1 cells were infected with lentiviruses expressing shRNAs against Slit2 or scrambled shRNA and were selected with 2 μg/ml puromycin. Four days after infection, 4 × 10^3^ cells were plated in soft agar. The soft agar cultures were comprised of two layers: a base layer (4 ml in a 60 mm dish; DMEM/10% fetal bovine serum/0.6% noble agar [A5431, Sigma-Aldrich]) and a cell layer (2 ml in a 60 mm dish; DMEM/10% fetal bovine serum/0.3% noble agar). Colonies were grown for two weeks and counted. Colonies (>50 cells) were scored by randomly counting 9 fields per dish. Sphere formation assays were done as described [36] using ultra-low attachment 6-well plates (Corning; seeding density 1 × 10^4^ cells/well) and DMEM/F-12 medium supplemented with B27 (17504044, Gibco), human recombinant epidermal growth factor (20 ng/ml) (100–15, Peprotech), and basic fibroblast growth factor (20 ng/ml) (100-18B, Peprotech). Spheres were grown for two weeks and counted.

### GST pull-down assay

Cells were lysed in TNE buffer (10 mM Tris, pH 7.4, 150 mM, NaCl, 1% NP-40, 1 mM EDTA, and protease inhibitors). The active form of cdc42, Rac, and Rho A was analyzed by pull-down of cell lysate with GST-PAK1 (which selectively binds active cdc42 and active Rac) and GST-RBD (which selectively binds active Rho A) followed by immunoblotting for cdc42, Rac, and Rho as described [37].

### Statistical analysis

Statistical analyses were performed with Prism (GraphPad Software) with a two-tailed Student’s *t* test. Data are expressed as mean ± SEM. The results were considered significant when *p* < 0.05.
